# Venomous snake bites: clinical diagnosis and treatment

**DOI:** 10.1186/s40560-015-0081-8

**Published:** 2015-04-01

**Authors:** Toru Hifumi, Atsushi Sakai, Yutaka Kondo, Akihiko Yamamoto, Nobuya Morine, Manabu Ato, Keigo Shibayama, Kazuo Umezawa, Nobuaki Kiriu, Hiroshi Kato, Yuichi Koido, Junichi Inoue, Kenya Kawakita, Yasuhiro Kuroda

**Affiliations:** Emergency Medical Center, Kagawa University Hospital, 1750-1 Ikenobe, Miki, Kita, Kagawa, 761-0793 Japan; The Japan Snake Institute, Yabuzuka 3318, Ota, Gunma, 379-2301 Japan; Department of Emergency Medicine, Graduate School of Medicine, University of the Ryukyus, 207, Uehara, Nishihara, Okinawa, 903-0215 Japan; Department of Bacteriology II, National Institute of Infectious Disease, Gakuen 4-7-1, Musashimurayama, Tokyo, 208-0011 Japan; Okinawa Prefectural Institute of Health and Environment, 2085 Ozato, Ozato, Nanjo, Okinawa, 901-1202 Japan; Department of Immunology, National Institute of Infectious Disease, Toyama 1-23-1, Shinjuku, Tokyo, 162-8640 Japan; Department of Emergency and Critical Care Medicine, Tokai University School of Medicine, 143 Shimokasuya, Isehara, Kanagawa, 259-1193 Japan; Division of Critical Care Medicine and Trauma, National Hospital Organization Disaster Medical Center, 3256 Midoricho, Tachikawa, Tokyo, 190-0014 Japan; Division of Critical Care Medicine and Trauma, Yamanashi Prefectural Central Hospital, 1-1-1 Fujimicho, Kofu, Yamanashi, 400-8506 Japan

**Keywords:** Mamushi, Habu, Yamakagashi, Antivenom

## Abstract

Snake bites are life-threatening injuries that can require intensive care. The diagnosis and treatment of venomous snake bites is sometimes difficult for clinicians because sufficient information has not been provided in clinical practice. Here we review the literature to present the proper management of bites by mamushi, habu, and yamakagashi snakes, which widely inhabit Japan and other Asian countries. No definite diagnostic markers or kits are available for clinical practice; therefore, definitive diagnosis of snake-venom poisoning requires positive identification of the snake and observation of the clinical manifestations of envenomation. Mamushi (*Gloydius blomhoffii*) bites cause swelling and pain that spreads gradually from the bite site. The platelet count gradually decreases due to the platelet aggregation activity of the venom and can decrease to <100,000/mm^3^. If the venom gets directly injected into the blood vessel, the platelet count rapidly decreases to <10,000/mm^3^ within 1 h after the bite. Habu (*Protobothrops flavoviridis*) bites result in swelling within 30 min. Severe cases manifest not only local signs but also general symptoms such as vomiting, cyanosis, loss of consciousness, and hypotension. Yamakagashi (*Rhabdophis tigrinus*) bites induce life-threatening hemorrhagic symptoms and severe disseminated intravascular coagulation with a fibrinolytic phenotype, resulting in hypofibrinogenemia and increased levels of fibrinogen degradation products. Previously recommended first-aid measures such as tourniquets, incision, and suction are strongly discouraged. Once airway, breathing, and circulation have been established, a rapid, detailed history should be obtained. If a snake bite is suspected, hospital admission should be considered for further follow-up. All venomous snake bites can be effectively treated with antivenom. Side effects of antivenom should be prevented by sufficient preparation. Approved antivenoms for mamushi and habu are available. Yamakagashi antivenom is used as an off-label drug in Japan, requiring clinicians to join a clinical research group for its use in clinical practice.

## Introduction

Throughout the world, snake bites remain life-threatening injuries [1-4], sometimes requiring intensive care [5]. Similar to malaria, dengue hemorrhagic fever, tuberculosis, and parasitic diseases, the risk of snake bite is always present [1]. In 2009, the World Health Organization (WHO) added snake bites to the list of neglected tropical diseases, which includes dengue hemorrhagic fever, cholera, and Japanese encephalitis. The mortality associated with snake bites is much greater than that of other neglected tropical diseases [1]. Moreover, the 2014 dengue fever outbreak in Tokyo, Japan, was promoted by climate change and intensive interaction between people; these factors may thus contribute to outbreaks of other tropical diseases in the future.

Venomous snakes of the same genus as mamushi (*Gloydius*), habu (*Protobothrops*), and yamakagashi (*Rhabdophis*) inhabit Japan and other Asian countries [6-8]. The incidence of bites by these venomous snakes is reported as approximately 1,000 cases with 10 deaths annually for mamushi (*Gloydius blomhoffii*) [9], 100 cases annually for habu (*Protobothrops flavoviridis*) [10], and 34 cases with 4 deaths over the past 40 years for yamakagashi (*Rhabdophis tigrinus*) [6].

The diagnosis and treatment of venomous snake bites is sometimes difficult for clinicians because sufficient information, including the administration of antivenom therapy, has not been provided in clinical practice [6,11]. Here we clarify the proper management of bites by mamushi, habu, and yamakagashi, including snake characteristics, venom activity and symptoms, clinical diagnosis, and treatment.

## Review

### Snake characteristics

#### *Mamushi* (*G. blomhoffii*)

**Figure 1:**
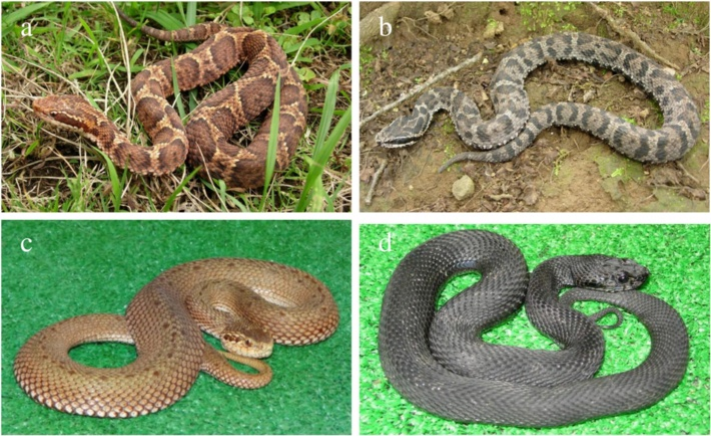
**Color variation in mamushi. (a)** Common color; (**b, c)** color variations; (**d)** melanistic variant. Photographs courtesy of the Japan Snake Institute.

**Figure 2:**
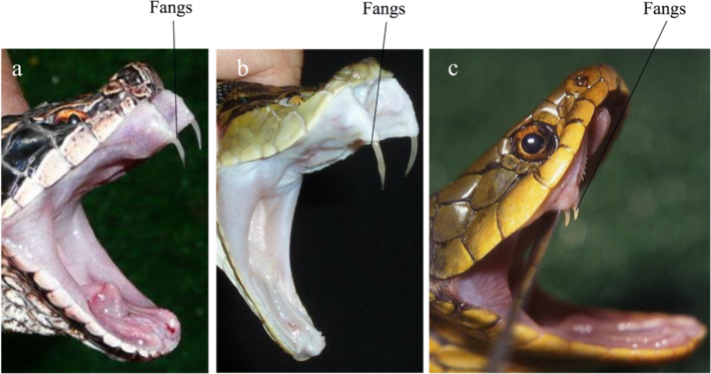
**Locations of fangs in mamushi, habu, and yamakagashi snakes. (a)** Mamushi fangs are about 5 mm long, with very thin tips. The snakes often have two fangs on each side; **(b)** habu fangs are 1.5 to 2 cm long; **(c)** yamakagashi fangs are only about 2 mm long and are located slightly back in the mouth. Photographs courtesy of the Japan Snake Institute **(a, c)** and the Okinawa Prefectural Institute of Health and Environment **(b)**.

Mamushi is a pit viper that is seen in a wide variety of colors (Figure 1). As mamushi is a small snake (about 60 cm), its attack range is only about 30 cm [11]. The fangs are about 5 mm long, with very thin tips (Figure 2a). This snake lives near rivers, ponds, and paddy fields and is active in the daytime in spring and autumn and at night in the summer. In Japan, *G. blomhoffii* is seen from Kyushu to Hokkaido, and the distinct species *Gloydius tsushimaensis* (Tsushima Mamushi) is found on Tsushima island, Nagasaki.

#### *Habu* (*P. flavoviridis*)

**Figure 3:**
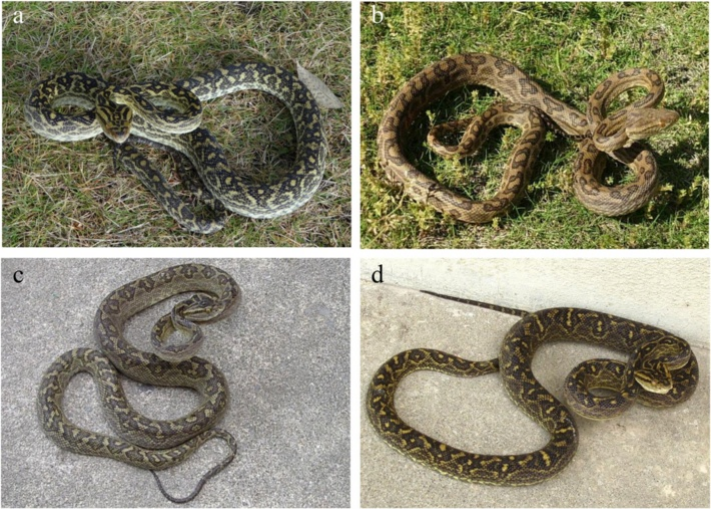
**Color variations in habu from different geographical locations.** Habu from **(a)** Amami Oshima; **(b)** Tokunoshima; and **(c, d)** Okinawa. Photographs courtesy of the Okinawa Prefectural Institute of Health and Environment.

Five types of pit vipers inhabit Okinawa and Amami. Habu, one of these pit vipers, varies in color by region (Figure 3). Even though this nocturnal snake is not active in the daytime, many people are bitten when disturbing snakes while farming. At night, this snake comes out in search of food near houses, sometimes entering them. Accidents often occur during handling. Habu snakes often climb trees. Habu is the most dangerous of these three snakes because it is large, reaching up to 2 m in length, and is the most aggressive. Habu fangs are tubular and 1.5–2 cm in length (Figure 2b). Dry bites can occur because the venom-releasing pore of the habu snake is located approximately 0.1 cm from the tip of the venom fang [12].

#### *Yamakagashi* (*R. tigrinus*)

**Figure 4:**
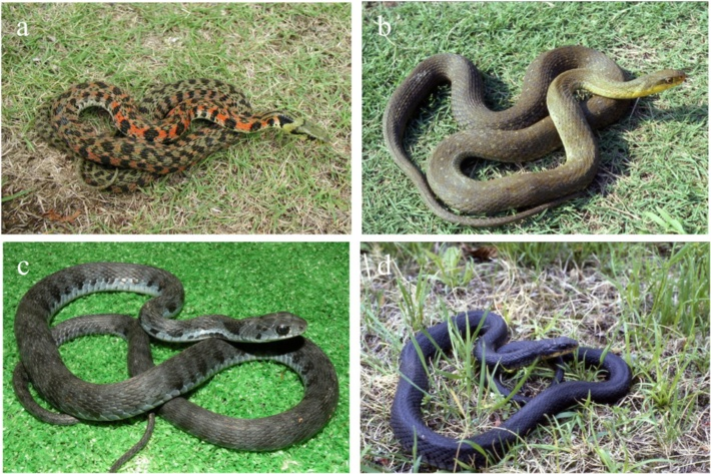
**Color variation in yamakagashi from different geographical locations.** Yamakagashi from **(a)** Kanto and Tohoku; **(b)** Chubu and Kinki; and **(c)** Chugoku and Sikoku; **(d)** melanistic variant. Photographs courtesy of the Japan Snake Institute.

Yamakagashi is a rear-fanged venomous snake that lives near rivers, ponds, and paddy fields, the same habitat as mamushi. Snakes of the same genus, such as *Rhabdophis lateralis* and *Rhabdophis subminiatus*, are distributed throughout Russia and Asia [13,14]. Yamakagashi grows to about 1 m in the plains and 1.5 m in the hills and mountains. The color varies by region (Figure 4). The larger snakes have short, 2-mm long fangs located slightly back from the front of the mouth. Like viper fangs, the fangs of yamakagashi are not tubular, and the venom gland duct opens at the base of the fang (Figure 2c). Because yamakagashi fangs are not grooved, envenomation does not occur in most bites; therefore, this snake has long been considered non-venomous [13,15].

### Venom activity and clinical symptoms

#### *Mamushi* (*G. blomhoffii*)

Several enzymes, including a protease, phospholipase A2 (PlA2), and bradykinin-releasing-enzyme are contained in the mamushi venom [16]. The effects of these enzymes are described in Table 1. Local pain and swelling are the main symptoms at the bite site; subcutaneous bleeding and blisters are sometimes observed. The swelling and pain spread gradually from the bite site (Table 2). Most patients are bitten on the hand or foot, but the spread of swelling to the trunk is often observed [17].

**Table 1:** **Enzymes in the snake venoms**

**Enzyme**	**Mamushi**	**Habu**	**Yamakagashi**
Non-hemorrhagic metalloproteinase	+	+	+
HR1	+	+	
HR2	+	+	
Phospholipase A2	+	+	
TAME esterase	+	+	
L-amino acid oxidase	+	+	
Hyaluronidase	+	+	
Phosphodiesterase	+	+	
Phosphomonoesterase	+	+	
ATPase	+	+	
5′-nucleotidase	+	+	
Endonuclease	+	+	
NAD-nucleosidase	+	±	
Alginine ester hydrolase	+	+	
Thrombin-like enzyme	+	+	±
Endopeptidase	+	+	
Bradykinin-releasing enzyme	+	+	

+ indicates the presence of the enzyme, − indicates the absence of the enzyme, blank space indicates unknown whether the venom contains the given enzyme, *HR 1* hemorrhagic factor-1, *HR-2* hemorrhagic factor-2, *TAME* p-toluenesulfonyl-L-arginine methyl ester, *ATPase* adenosine triphosphatase, *NAD* nicotinamide adenine dinucleotide.

**Table 2:** **Typical symptoms and laboratory data to be evaluated**

	**Mamushi**	**Habu**	**Yamakagashi**
Typical symptoms	Local pain, swelling, severely decreased platelet count^a^, diplopia, blurred vision, nausea^b^, vomiting^b^, stomachache^b^, diarrhea^b^, cyanosis^b^	Local swelling, necrosis, bleeding at the bite site, vomiting^b^, cyanosis^b^, loss of consciousness^b^, hypotension^b^, compartment syndrome	Nasal bleeding, gum bleeding, bleeding from the bite site, headache^b^
Laboratory data to be evaluated routinely	CBC, CK, BUN, Cre, Na, K, Cl, Fibrinogen, FDP, d-dimer, PT, APTT		
Typical laboratory findings	CK↑		FDP >100 μg/mL
	Plt <10,000/μL^a^		Fibrinogen <100 mg/dL
Laboratory data to be evaluated additionally	Myoglobin, CK-MB	Myoglobin	AT-III, TAT, PIC

*CBC* complete blood count, *Plt* platelet count, *CK* creatine kinase, *BUN* blood urea nitrogen, *Cre* creatinine, *FDP* fibrinogen degradation products, *PT* prothrombin time, *APTT* activated partial thromboplastin time *AT-III* antithrombin III, *TAT* thrombin-antithrombin III complex, *PIC* plasmin-α2-plasmin inhibitor complex.

^a^Venom is injected into the blood vessel directly.

^b^In severe cases.

With severe swelling, hypotension can occur. In these cases, increased levels of creatine phosphokinase (CPK) and blood myoglobin due to rhabdomyolysis are remarkable and can cause acute renal failure [11,17]. In addition to hypotension, renal hemorrhage and direct action of the venom on the kidney can cause acute renal failure. In severe cases, the plasma potassium level can increase due to muscle tissue damage and metabolic acidosis, causing cardiac arrest shortly after the bite [18,19]. A rise in the level of the CPK isozyme cardiac muscle conformer (MB) and necrosis of the myocardium have been reported, which may be due to the direct action of venom on the cardiac muscle [20].

As the venom is absorbed from the bite site, the platelet count gradually decreases due to the platelet aggregation activity of the venom, sometimes decreasing to <100,000/mm^3^ [21]. Cases in which the platelet count rapidly decreases to <10,000/mm^3^ within 1 h after the bite are often seen [22]. The venom is thought to be injected directly into the blood vessel during the bite, as the tips of the mamushi fangs are very thin. The platelet aggregation and hemorrhagic activities are very strong, causing ecchymosis and gastrointestinal bleeding. However, even in severe cases, little change in prothrombin time (PT), activated partial thromboplastin time (APTT), or fibrinogen levels is observed [11]. The vasodilatation activity of the venom is strong, sometimes causing hypotension [23].

The venom contains small amounts of neurotoxin, which causes diplopia, blurred vision, and a divergent squint due to action on the nervus oculomotorius, but respiratory muscle paralysis is not seen. These ocular symptoms remit within several days to about 2 weeks [24].

#### *Habu* (*P. flavoviridis*)

The toxicity of habu venom is about half that of mamushi venom, but the amount of habu venom is approximately 10 times that of mamushi venom. Since habu venom contains many enzymes similar to those found in mamushi venom (except the neurotoxin), a similar range of symptoms are observed in patients with habu bites (Table 1). Habu venom causes extreme local swelling, necrosis, and bleeding at the bite site (Table 2). Most habu bites start to swell within 30 min after the bite [7]. In addition, severe cases manifest not only with local signs but also with general symptoms such as vomiting, cyanosis, loss of consciousness, and hypotension. Habu bites frequently cause compartment syndrome (CS) because of the large volume of venom injected, regardless of its lower toxicity compared to mamushi venom. In addition, following the bite, patients tend to bind the wound excessively tightly due to fear of the venom spreading to the whole body, thereby exacerbating CS. Thus, many cases of CS are reported following habu bites.

#### *Yamakagashi* (*R. tigrinus*)

Yamakagashi venom (metalloproteinase) has strong blood coagulation activity, with a prothrombin-activating effect and a weak thrombin-like effect [25]. Once yamakagashi venom enters the blood, it activates prothrombin continuously, causing excessive coagulation. Disseminated fibrin formation ensues, and fibrinolysis is activated, resulting in hypofibrinogenemia and increased levels of fibrinogen degradation products (FDP) [5]. This venom induces life-threatening hemorrhagic symptoms and severe disseminated intravascular coagulation (DIC) with a fibrinolytic phenotype that is typically observed in patients with acute, severe blunt trauma [26], acute leukemia (particularly in acute promyelocytic leukemia) [27], and massive obstetric hemorrhage [28]. DIC progresses to acute renal failure due to the obstruction of glomeruli by thrombi. Because the fangs of this snake are very short, the venom is injected subcutaneously or intradermally. However, pain, swelling, and inflammation are minimal at the bite site because the venom does not act on the tissues directly. The typical symptom is hemorrhage, including nasal bleeding, gum bleeding, and bleeding from the bite site (Table 2). In severe cases, headache is also a characteristic symptom [5].

### Diagnosis

There are no definite diagnostic markers or kits available in clinical practice; therefore, definitive diagnosis of snake-venom poisoning requires positive identification of the snake and observation of the clinical manifestations of envenomation [3]. On initial assessment, CBC, BUN, Cre, Na, K, Cl, CK, and coagulation markers (fibrinogen, FDP, d-dimer, PT, and APTT) should be examined (Table 2).

#### *Mamushi* (*G. blomhoffii*)

Because mamushi hide in the grass and fallen leaves, identification is difficult, even in the daytime. Patients usually feel only a pain similar to that of a splinter because the fangs are about 5 mm long and very thin. Thus, patients and physicians often mistake this bite for an insect bite or sting, especially when bitten at night [11]. The mamushi bite usually leaves two very small wounds that are 1 cm apart [11]. These snakes often have two fangs on each side; therefore, three or four fang marks are often observed. As small bite marks may be difficult to observe, diagnosis by bite wounds alone is difficult [11]. In many cases, blood test data do not change for several hours after the bite. If symptoms such as swelling are seen, it is necessary to perform frequent blood tests. With increased swelling, CK and blood myoglobin levels rise, followed by a rise in BUN and creatinine levels. A remarkable rise in myoglobin level is an indicator for the diagnosis of mamushi bite and suggests the risk of acute renal failure.

In cases where venom was injected into the blood vessel directly, platelet counts rapidly decrease to <10,000/mm^3^ but fibrinogen levels do not decrease [22]. Such cases are difficult to diagnose because the local symptoms are mild. However, if swelling, hypotension, or ocular symptoms such as double vision or squint are observed, the identity of the snake is most likely mamushi. In severe cases, nausea, vomiting, stomachache, diarrhea, cyanosis, and tachycardia are sometimes observed.

Grade classification for mamushi bites is clinically used to determine the severity of injuries as follows [17,29]: Grade I, redness and swelling around the bitten area; Grade II, redness and swelling of the wrist or foot joint; Grade III, redness and swelling of the elbow or knee joint; Grade IV, redness and swelling of the whole extremity; and Grade V, redness and swelling in parts beyond the extremity or exhibiting systemic symptoms.

#### *Habu* (*P. flavoviridis*)

There are no standardized diagnostic or severity criteria for habu bites. Local swelling may help determine whether the patient was bitten by a habu. Because habu bites result in swelling within 30 min, the circumference of the affected limb may be one indicator of severity. Twenty percent of habu bites are dry. This incidence is higher than that of bites by other snakes, such as the saw-scaled viper (*Echis carinatus*) with 8% dry bites and the rattlesnake in Central California with 10.9% dry bites [30-32]. While most dry bite cases do not require admission, Levine recommends repeating laboratory test within 6 h [4].

#### *Yamakagashi* (*R. tigrinus*)

Yamakagashi bites have been diagnosed based on detailed descriptions of snakes by patients and hemorrhagic symptoms including severe hypofibrinogenemia (<100 mg/dL) [6]. In one study, about 80% of the reported patients developed persistent bleeding from the bite site on admission [5]. DIC with a fibrinolytic phenotype develops early; therefore, evaluating the DIC score is mandatory for the diagnosis of this injury [33]. Assessment of the levels of antithrombin III (AT-III), thrombin-antithrombin III complex (TAT) and plasmin-α2-plasmin inhibitor complex (PIC) may help to evaluate the clinical condition.

### Treatment

Previously recommended first-aid measures are strongly discouraged [3]. The use of tight ligatures and arterial tourniquets in the first-aid treatment of snakebite has been universally condemned by modern snakebite experts due to the increase of potential adverse effects and the lack of effectiveness [34-36]. No human study has shown the efficacy of incision and suction as a first-aid tool with regard to improvement of survival or outcome [37].

Once airway, breathing, and circulation have been established, a rapid, detailed history should be obtained [3,37]. If a snake bite is suspected, hospital admission should be considered for further follow-up.

#### Antivenom therapy

Snake antivenoms are manufactured by immunizing horses against unbound venom. Antivenom treatment is the definitive therapy, but not all cases warrant such therapy (Table 3). Antivenom is administered intravenously to achieve rapid onset of action [7,11]. Subcutaneous or intramuscular injection for the purpose of avoiding side effects is not recommended.

**Table 3:** **Indication and incidence of side effects in antivenom**

	**Mamushi**	**Habu**	**Yamakagashi**
Indication	Mamushi grade ≧ III	N/A	Fibrinogen <100 mg/dL
Side effects			
Anaphylaxis	2.4%–9.0%	11%	0%
Serum sickness disease	N/A	24.2%	N/A

*N/A* not applicable.

Because snakes inject the same amount of venom into adults and children, the same dose/volume of antivenom must be administered to children.

Preparedness for anaphylaxis should be considered when administering the antivenom. Premedication with an antihistamine and/or epinephrine should be used when the perceived benefit is greater than the risk of adverse effects [5]. As for the use of hydrocortisone as premedication for snake antivenom, the efficacy has not been determined [38].

Another major adverse effect of antivenom is serum sickness disease, which usually occurs 4–10 days after antivenom administration [39]. Rashes, itching, joint pain, fever, lymphadenopathy, malaise, and renal failure are typical symptoms [39,40]. Serum sickness disease is the prototypical type III hypersensitivity reaction, involving excessive immune complex formation [41]. Although many patients have mild symptoms, the reaction can lead to multiple organ failure. Such severe reactions most often occur in patients with severe snakebites that require large amounts of antivenom. Systematic corticosteroids are the main treatment of choice, starting at a dose of 60 mg per day and tapering over 2 weeks to avoid rebound [3,42]. Plasmapheresis is used to obtain rapid effectiveness, particularly in severe cases [43,44].

Mamushi and habu antivenom are approved drugs, whereas yamakagashi antivenom is used as an off-label drug in Japan. Therefore, clinicians are required to join a clinical research group to use yamakagashi antivenom in clinical practice [6].

### Efficacy of antivenom

#### *Mamushi* (*G. blomhoffii*)

Studies have evaluated the efficacy of antivenom and cepharanthine (CEP) in a single-center cohort study [45,46]. Makino et al. evaluated 114 cases and reported that patients administered antivenom had a significantly shorter hospital stay than those administered CEP (*p* < 0.01). However, in severe cases (grades of mamushi bites IV/V), the percentage of patients administered antivenom was higher than that of patients administered CEP (50% vs. 33%, *p* = 0.06) [45]. In contrast, Kochi et al*.* evaluated 50 cases and reported that patients administered antivenom had a significantly longer hospital stay than those administered CEP because of the greater severity of cases in the antivenom group [46]. Thus, evaluating the efficacy of antivenom and CEP without adjusting for the severity of mamushi bites limited these studies [45,46].

**Figure 5:**
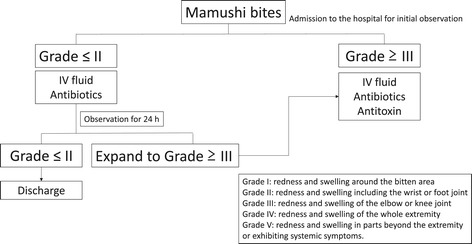
**The clinical decision algorithm for Mamushi bites.** IV fluid, intravenous fluid administration.

Until 1990, antivenom was most often administered subcutaneously or intramuscularly to avoid adverse reactions. Due to its slow absorption in the human body, mamushi antivenom was mistakenly assumed to be ineffective by clinical doctors [47]. Intravenous administration of antivenom had started in the 1990s, and proper re-evaluation of the antivenom was expected. Hifumi et al. conducted large, multi-center, population-based studies [17], reporting 234 mamushi bites. Among the severe cases (grades III/IV/V), patients administered antivenom had a significantly shorter hospital stay than those administered CEP (*p* = 0.024). In contrast, for the mild cases (grades I/II), there was no significant difference in the duration of hospital stay between the two groups (*p* = 0.77). Therefore, the authors concluded that antivenom is effective in shortening the duration of hospital stay for patients with severe mamushi bites [17]. We propose a new clinical decision algorithm for mamushi bites as shown in Figure 5. We recommend antivenom administration in patients with mamushi grade ≧III on the basis of our previously reported data [17].

#### *Habu* (*P. flavoviridis*)

No definitive indication for the use of antivenom has been provided in clinical practice. Although antivenom is considered effective following habu bites, there are no large-scale studies of the prognosis. Okinawa prefecture is known to have a large population of habu, and the rate of antivenom use is high. There have been no deaths from habu bites in the last 10 years in this area (2004–2013, no deaths in 551 cases) [48]. However, between 1965 and 1969, there were approximately 24 deaths among 1,770 cases in Okinawa due to the lack of antivenom [48]. Therefore, antivenom therapy is currently considered useful for habu bites [49,50].

#### *Yamakagashi* (*R. tigrinus*)

Hifumi et al. conducted a retrospective survey analyzing data from 34 patients (19 of whom were treated with antivenom) between 1973 and 2013 [5]. Univariate analysis revealed no significant difference in baseline characteristics and laboratory data between those treated with and without antivenom. Hospital mortality was significantly lower in patients treated with antivenom than in those treated without (0% vs. 26.7%; *p* = 0.03). Moreover, the number of patients with renal failure requiring hemodialysis was significantly lower among those treated with antivenom (5.3% vs. 40.0%; *p* = 0.03).

Therefore, antivenom is a specific, definitive, and effective treatment. Administration of yamakagashi antivenom following bites can lead to complete clinical recovery without progression to multiple organ dysfunction syndrome (MODS), even in the presence of severe DIC. Thus, antivenom effectively treats the acute symptoms and can prevent disease progression. Fibrinogen levels <100 mg/dL are considered appropriate for antivenom administration in clinical practice [13,51].

### Antivenom side effects

#### *Mamushi* (*G. blomhoffii*)

A recent national survey reported that the incidence of adverse reactions to antivenom was 2.4%–9.0%, including mild cases [9,17].

#### *Habu* (*P. flavoviridis*)

Miyagi reported that habu antivenom induced early allergic reactions in approximately 11% and serum sickness disease in approximately 24.2% of patients [7]. The reason this antivenom has higher rates of allergic reactions than the other two antivenoms produced using horses remains unknown.

#### *Yamakagashi* (*R. tigrinus*)

Although the number of the included patients is small (34 cases) to make any comprehensive assessment, the initial anaphylactic reaction rate (including severe reactions) was zero [5,6].

### Other treatments

#### *Mamushi* (*G. blomhoffii*)

CEP, a biscoclaurine (bisbenzylisoquinoline) amphipathic alkaloid isolated from *Stephania cepharantha* Hayata, has been proposed as a possible alternative therapy to antivenom because it lessens the inflammation and pain caused by snake bites [9]. CEP and other extracts from the same plant are widely used in clinical practice (primarily in Japan) to treat a variety of acute and chronic diseases such as alopecia areata [52], radiotherapy-induced leucopenia [53], malaria [54], and septic shock [55]. However, CEP does not have the ability to neutralize circulating venom [56]; therefore, CEP should not be used instead of antivenom for treating mamushi bites (Figure 5). A previously proposed clinical decision algorithm for mamushi bites (supported by the pharmaceutical company) recommends the routine use of CEP [57]; however, the routine use of CEP is clearly unnecessary considering its limited effectiveness.

Because no cases of tetanus associated with mamushi bites have been reported, routine use of tetanus toxoid in patients with mamushi bites is not recommended (Figure 5).

#### *Habu* (*P. flavoviridis*)

Because myonecrosis and CS are often observed, our goal in treatment is not only to save lives but to improve functional outcomes [58,59]. Habu bites caused 14 cases of CS in 2009 [60]. Fasciotomy is required when the compartment pressure reaches 30 mmHg. However, when pressures only moderately exceeds 30 mmHg, some people advocate management with further antivenom, elevation, and reassessment within a few hours; in such cases, fasciotomy is only considered if pressures fail to decrease within several hours [3,32]. This protocol may be the preferable option for mildly symptomatic patients. The initial use of intravenous fluids is also effective for improving circulatory dysfunction and preventing renal dysfunction caused by CS.

#### *Yamakagashi* (*R. tigrinus*)

Yamakagashi bites induce DIC, for which heparin has been used [5]. However, heparin use is contraindicated considering the pathophysiology of DIC involving fibrinolysis. Although other adjunct DIC treatments, such as protease inhibitors and fresh frozen plasma (FFP), are clinically used, the only definitive therapy available is antivenom.

## Conclusions

In this review, we have provided information to clarify the clinical diagnosis of snake bites. Antivenom therapy is warranted for venomous snake bites.

## References

[1] Williams D, Gutierrez JM, Harrison R, Warrell DA, White J, Winkel KD (2010). The Global Snake Bite Initiative: an antidote for snake bite. Lancet.

[2] Warrell DA (2010). Snake bite. Lancet.

[3] Gold BS, Dart RC, Barish RA (2002). Bites of venomous snakes. N Engl J Med.

[4] Kitchens CS, Van Mierop LH (1987). Envenomation by the Eastern coral snake (Micrurus fulvius fulvius). A study of 39 victims. JAMA.

[5] Hifumi T, Sakai A, Yamamoto A, Murakawa M, Ato M, Shibayama K (2014). Effect of antivenom therapy of Rhabdophis tigrinus (Yamakagashi snake) bites. J Intensive Care.

[6] Hifumi T, Sakai A, Yamamoto A, Murakawa M, Ato M, Shibayama K (2014). Clinical characteristics of yamakagashi (Rhabdophis tigrinus) bites: a national survey in Japan, 2000–2013. J Intensive Care.

[7] Miyagi Y (2007). Habu-bite. Jpn J Toxicol.

[8] Qian XH, Ma L (1991). Fibrinolytic enzyme from Agkistrodon halys brevicaudus (Korean mamushi) snake venom. Toxicon.

[9] Hifumi T, Yamamoto A, Morokuma K, Ogasawara T, Kiriu N, Hasegawa E (2011). Surveillance of the clinical use of mamushi (Gloydius blomhoffii) antivenom in tertiary care centers in Japan. Jpn J Infect Dis.

[10] Annual incidence of Habu bites. http://www.eikanken-okinawa.jp/seitaiG/habu/habu6.htm

[11] Sakai A (2013). Diagnosis and treatment of snakebite by Mamushi and Yamakagashi. Chudoku Kenkyu.

[12] Yoshida C (1979). Habu and Human.

[13] Morokuma K, Kobori N, Fukuda T, Uchida T, Sakai A, Toriba M (2011). Experimental manufacture of equine antivenom against yamakagashi (Rhabdophis tigrinus). Jpn J Infect Dis.

[14] Silva A, Hifumi T, Sakai A, Yamamoto A, Murakawa M, Ato M (2014). Rhabdophis tigrinus is not a pit viper but its bites result in venom-induced consumptive coagulopathy similar to many viper bites. J Intensive Care.

[15] Hifumi T, Murakawa M, Sakai A, Ginnaga A, Yamamoto A, Kato H, Koido Y, et al. A case of potentially fatal coagulopathy secondary to yamakagashi (Rhabdophis tigrinus) bites that completely recovered with antivenom administration. Acute Med Surg. In press. 10.1002/ams2.69 PMC5667206 29123706

[16] Suzuki T (1970). Studies on snake venom enzymes, centering around Agkistrodon haly blomhoffii veom. Snake.

[17] Hifumi T, Yamamoto A, Morokuma K, Okada I, Kiriu N, Ogasawara T (2013). Clinical efficacy of antivenom and cepharanthine for the treatment of Mamushi (Gloydius blomhoffii) bites in tertiary care centers in Japan. Jpn J Infect Dis.

[18] Hamasaki T, Okano K (1986). Twenty cases of snake (mamushi) bite. Tottori Med J.

[19] Yamashita M, Suehiro K, Otani M (1986). A death case of mamushi bite. Shimane Igaku.

[20] Gyotoku T, Kiyoi K (2002). A death case of mamushi bite. Nishinihon J Drmatology.

[21] Tachibana K, Itoh S (1986). A case report of multiple organ failure (MOF) caused by Mamushi bite. JJAAM.

[22] Fujita M, Yamashita S, Kawamura Y (2005). Viper (Agkistrodon halys blomhoffii “Mamushi”) bite with remarkable thrombocytopenia. JJAAM.

[23] Ishida T, Koyama T (1995). Viper (Agkistrodon halys “Mamushi”) bite with shock - bleeding diathesis and serious hematemesis. JJAAM.

[24] Mori K, Takeyama Y (1998). Two cases of severe mamushi bite with ocular signs. Japn J Toxicol.

[25] Komori K, Konishi M, Maruta Y, Toriba M, Sakai A, Matsuda A (2006). Characterization of a novel metalloproteinase in Duvernoy's gland of Rhabdophis tigrinus tigrinus. J Toxicol Sci.

[26] Gando S, Tedo I, Kubota M (1992). Posttrauma coagulation and fibrinolysis. Crit Care Med.

[27] Barbui T, Falanga A (2001). Disseminated intravascular coagulation in acute leukemia. Semin Thromb Hemost.

[28] McLintock C, James AH (2011). Obstetric hemorrhage. J Thromb Haemost.

[29] Sakio H, Yokoyama K, Uchida T (1985). Mamushi (viper) bite in Kensei General Hospital. Rinsho Geka.

[30] Kularatne SA, Sivansuthan S, Medagedara SC, Maduwage K, de Silva A (2011). Revisiting saw-scaled viper (Echis carinatus) bites in the Jaffna Peninsula of Sri Lanka: distribution, epidemiology and clinical manifestations. Trans Roy Soc Trop Med Hyg.

[31] Spano S, Macias F, Snowden B, Vohra R (2013). Snakebite Survivors Club: retrospective review of rattlesnake bites in Central California. Toxicon.

[32] Levine M, Ruha AM, Graeme K, Brooks DE, Canning J, Curry SC (2011). Toxicology in the ICU: part 3: natural toxins. Chest.

[33] Asakura H (2014). Classifying types of disseminated intravascular coagulation: clinical and animal models. J Intensive Care.

[34] Amaral CF, Campolina D, Dias MB, Bueno CM, Rezende NA (1998). Tourniquet ineffectiveness to reduce the severity of envenoming after Crotalus durissus snake bite in Belo Horizonte, Minas Gerais. Brazil Toxicon.

[35] Pugh RN, Theakston RD (1987). Fatality following use of a tourniquet after viper bite envenoming. Ann Trop Med Parasitol.

[36] Watt G, Padre L, Tuazon ML, Theakston RD, Laughlin LW (1988). Tourniquet application after cobra bite: delay in the onset of neurotoxicity and the dangers of sudden release. Am J Trop Med Hyg.

[37] Hall EL (2001). Role of surgical intervention in the management of crotaline snake envenomation. Ann Emerg Med.

[38] Soh SY, Rutherford G (2006). Evidence behind the WHO guidelines: hospital care for children: should s/c adrenaline, hydrocortisone or antihistamines be used as premedication for snake antivenom?. J Trop Ped.

[39] Lundquist AL, Chari RS, Wood JH, Miller GG, Schaefer HM, Raiford DS (2007). Serum sickness following rabbit antithymocyte-globulin induction in a liver transplant recipient: case report and literature review. Liver Transpl.

[40] Davies KA, Mathieson P, Winearls CG, Rees AJ, Walport MJ (1990). Serum sickness and acute renal failure after streptokinase therapy for myocardial infarction. Clin Exp Imm.

[41] Kanji S, Chant C (2010). Allergic and hypersensitivity reactions in the intensive care unit. Crit Care Med.

[42] Bonds RS, Kelly BC (2013). Severe serum sickness after H1N1 influenza vaccination. Am Journal Med Sci.

[43] Chao YK, Shyur SD, Wu CY, Wang CY (2001). Childhood serum sickness: a case report. J Microbiol Immunol Infect.

[44] Boothpur R, Hardinger KL, Skelton RM, Lluka B, Koch MJ, Miller BW (2010). Serum sickness after treatment with rabbit antithymocyte globulin in kidney transplant recipients with previous rabbit exposure. Am J Kid Dis.

[45] Makino M, Yurugi E, Abe J (1988). A study of 114 cases of pit viper bite—with special reference to the administration of antivenom. J Jpn Pract Surg Soc.

[46] Kochi K, Okita M, Ito T (1995). A study of 50 cases of mamushi bite. J Jpn Pract Surg Soc.

[47] Sakai A (2001). Mamushi, Habu. Yamakagashi Rinsyoi.

[48] Uezu Y, Oshiro A, Terada K, Morine N. Venomous snakebite in Okinawa prefecture in 2014. Access date 2015/03/24 http://www.eikanken-okinawa.jp/seitaiG/habu/houkokusyo/H25houkoku.pdf

[49] Yamakawa M, Nozaki M, Hokama Z. Study of the effectiveness of HABU antivenom. Annual Report of Okinawa Prefectural Institute of Health and Environment. Access date 2015/03/24 http://www.pref.okinawa.lg.jp/site/hoken/eiken/syoho/documents/s10_102-115.pdf

[50] Nozaki M, Miyagi Y, Hokama Z (1978). Study of the effectiveness of antivenom for Okinawa habu (IX), Okinawa habu antivenom production research report (III).

[51] Dart RC, McNally J (2001). Efficacy, safety, and use of snake antivenoms in the United States. Ann Emer Med.

[52] Morita K, Nakamura M, Nagamachi M, Kishi T, Miyachi Y (2002). Seventeen cases of alopecia areata: combination of SADBE topical immunotherapy with other therapies. J Dermatol.

[53] Ohta TM, Morita K (1990). Effect of cepharanthin (sic) on radiotherapy induced leucopenia. Jpn J Clin Radiol.

[54] Chea A, Hout S, Bun SS, Tabatadze N, Gasquet M, Azas N (2007). Antimalarial activity of alkaloids isolated from Stephania rotunda. J Ethnopharm.

[55] Goto M, Zeller WP, Hurley RM (1991). Cepharanthine (biscoclaurine alkaloid) treatment in endotoxic shock of suckling rats. J Pharm Pharmacol.

[56] Ebisawa I, Sawai Y, Kawamura Y (1994). Some problems for Cepharanthine therapy to Maumushi bite. Jpn Med J.

[57] Flow chart for mamushi bites. http://www.aso-alkaloid.co.jp/04/4_4_81.htm

[58] Abubakar SB, Habib AG, Mathew J (2010). Amputation and disability following snakebite in Nigeria. Trop Doct.

[59] Laohawiriyakamol S, Sangkhathat S, Chiengkriwate P, Patrapinyokul S (2011). Surgery in management of snake envenomation in children. World J Pediatr.

[60] Le M (2009). 14 cases of compartment syndrome caused by Habu bite. J Jpn Assoc Surg Trauma.

